# Streamlining cVEP Paradigms: Effects of a Minimized Electrode Montage on Brain–Computer Interface Performance

**DOI:** 10.3390/brainsci15060549

**Published:** 2025-05-23

**Authors:** Milán András Fodor, Atilla Cantürk, Gernot Heisenberg, Ivan Volosyak

**Affiliations:** 1 Faculty of Technology and Bionics, Rhine-Waal University of Applied Sciences, 47533 Kleve, Germany; 2 Institute of Information Science, Technical University of Applied Sciences Cologne, 50678 Cologne, Germany

**Keywords:** brain–computer interface (BCI), BCI speller, code-modulated visual evoked potential (cVEP), EEG-based BCI, visual evoked potential (VEP), electrode reduction

## Abstract

(1) Background: Brain–computer interfaces (BCIs) enable direct communication between the brain and external devices using electroencephalography (EEG) signals, offering potential applications in assistive technology and neurorehabilitation. Code-modulated visual evoked potential (cVEP)-based BCIs employ code-pattern-based stimulation to evoke neural responses, which can then be classified to infer user intent. While increasing the number of EEG electrodes across the visual cortex enhances classification accuracy, it simultaneously reduces user comfort and increases setup complexity, duration, and hardware costs. (2) Methods: This online BCI study, involving thirty-eight able-bodied participants, investigated how reducing the electrode count from 16 to 6 affected performance. Three experimental conditions were tested: a baseline 16-electrode configuration, a reduced 6-electrode setup without retraining, and a reduced 6-electrode setup with retraining. (3) Results: Our results indicate that, on average, performance declines with fewer electrodes; nonetheless, retraining restored near-baseline mean Information Transfer Rate (ITR) and accuracy for those participants for whom the system remained functional. The results reveal that for a substantial number of participants, the classification pipeline fails after electrode removal, highlighting individual differences in the cVEP response characteristics or inherent limitations of the classification approach. (4) Conclusions: Ultimately, this suggests that minimal cVEP-BCI electrode setups capable of reliably functioning across all users might only be feasible through other, more flexible classification methods that can account for individual differences. These findings aim to serve as a guideline for what is currently achievable with this common cVEP paradigm and to highlight where future research should focus in order to move closer to a practical and user-friendly system.

## 1. Introduction

Brain–computer interfaces (BCIs) enable direct communication between the brain and external devices by translating neural activity into actionable commands, thereby bypassing conventional motor pathways. These systems typically rely on electroencephalography (EEG), a non-invasive neuroimaging technique that records electrical activity generated by the brain [1]. Over the past few decades, BCIs have gained significant attention in both research and clinical settings due to their potential to enhance human–computer interaction, restore lost motor functions, and facilitate assistive communication for individuals with severe motor impairments. By decoding neural responses associated with cognitive or sensory processes, BCIs can enable users to control external devices, interact with their environments, and communicate using brain activity alone. Despite considerable progress, challenges remain regarding the system reliability, usability, and accessibility of these systems in real-world applications [2].

Among various BCI paradigms, event-related potentials (ERPs) are widely used due to their strong correlation with external stimuli. A well-studied subclass of ERPs is the visual evoked potential (VEP), which is a neural response generated in reaction to visual stimuli. VEP-based BCIs leverage these visually evoked responses to infer user intent, such as where the person was looking on the screen. Compared to other BCI paradigms, VEP-based systems offer advantages such as high information transfer rate (ITR), intuitive operation, and non-invasiveness, making them particularly attractive for assistive communication [3,4].

Code-modulated visual evoked potential (cVEP) is a very effective VEP-based paradigm; it employs a stimulation pattern defined by a code sequence to elicit VEP responses [4,5]. In contrast to the more popular steady-state VEP (SSVEP) paradigm [3,6,7], which relies on a constant, periodic pattern to generate VEPs at a specific frequency, cVEP-based BCIs utilize code sequences—such as binary codes (e.g., m-sequences [8])—to define the visual stimulus. These sequences create a flickering pattern that elicits distinct neural responses, which can be classified using conventional methods such as those based on canonical correlation analysis (CCA) or machine learning-based classifiers. The cVEP stimulation is inherently non-constant and often pseudorandom due to the nature of the employed code patterns. In addition, the same code sequences are typically reused as stimuli for different classes by applying phase shifts. As a result, an extra layer of complexity is introduced to the signal processing and classification processes [5,9,10].

EEG stands out as the most practical and widely adopted method for measuring VEP responses, thanks to its non-invasive nature, excellent temporal resolution, and portability [1,11]. In VEP-based BCIs, electrode placement plays a crucial role in determining signal quality. One of the main ways to lower costs and the preparation time and even the discomfort that the BCI user feels is to use fewer electrodes. Reducing the number of electrodes in VEP-based BCIs has been shown to offer several practical advantages, including improved user comfort, shorter setup time, lower bandwidth requirements, and reduced hardware costs due to fewer electrodes and the potential use of a less advanced amplifier [3,6,12].

The major challenge associated with electrode reduction is the potential decline in classification accuracy due to the loss of spatial information [13]. Additionally, noise and movement artifacts already pose a challenge to the reliability of EEG-based BCIs [14]. Some studies also indicate increased variability in the location and strength of detectable cVEP responses [15], suggesting potential limitations due to individual differences. Furthermore, the classification pipelines for cVEP offer varying levels of flexibility and ease of retraining, which can influence the feasibility of a lower-electrode-count setup [5,14].

Utilizing minimal electrode montages is generally more feasible in SSVEP than in cVEP, since code-modulated VEPs elicit transient, broadband neural responses that engage a broader region of the visual cortex, whereas steady-state VEPs are more narrowly focused in time and space [14]. Studies of electrode placement and count in SSVEP-based BCIs indicate that, although reducing electrodes can affect classification performance, optimal configurations with only a few electrodes can be identified, although the precise layouts remain debated [12,13]. For the SSVEP paradigm, the strongest responses are typically observed over the occipital cortex (EEG sites O1, Oz, O2) and parietal regions [13], but this may be actually different for the cVEP BCI paradigm and needs further investigations.

Sixteen active [16] or passive [17] electrode montages have been shown to be a popular choice for achieving stable performance in cVEP-based BCIs. A recent study has assessed how increasing electrode density affects performance while accounting for target size. Sun et al. [18] compared online setups of 256, 128, 64, and 21 electrodes, and in offline analysis and simulation even down to 2 electrodes. In their study with 16 participants, they demonstrated that a nine-electrode configuration could still achieve over 90% accuracy in a 30-target system when stimuli were sufficiently large; however, their offline evaluation also revealed a drastic accuracy decline with single-digit electrode counts, but the study did not go into detail on the the measures of this.

Several studies have utilized low-count electrode setups. Some notable examples include the following: Thielen et al. [19] used eight non-invasive, water-based electrodes placed at Fz, T7, T8, POz, O1, Oz, O2, and Iz (10–20 system). Using their reconvolution classification method across nine participants, they achieved a mean ITR of 66.4 bits/min and an accuracy of 99.6%.

Wei et al. [20] realized a 48-target system with nine occipital–parietal electrodes (P3, Pz, P4, PO7, POz, PO8, O1, Oz, O2), and targets were decoded via CCA-based spatial filtering followed by template matching. In offline tests (12 subjects), per-group accuracy averaged 95.49% versus 92.85% across all 48. In a simulated online experiment (10 subjects), single-group accuracy was 94.9% and 48-target accuracy 91.7%, yielding ITRs of 135.6 bit/min and 181 bit/min, respectively.

Shirzhiyan et al. [21] evaluated a four-electrode system with an occipital–parietal montage (Pz, O1, Oz, O2) in a simulated online cVEP paradigm using standard CCA for classification. Despite the low channel count, they achieved mean accuracies above 91.7% across 44 targets, illustrating the potential of single-digit electrode setups with CCA-based classification.

Using only a single bipolar Oz–Pz electrode, Behboodi et al. [22] evaluated a six-target paradigm in 16 healthy volunteers with simple correlation-based template matching, achieving 70.1% accuracy.

Another study by Nezamfar et al. [23] demonstrated a working system with a single Oz electrode. They implemented a four-target cVEP application with ten healthy participants, achieving a mean accuracy of 92.6% and showcasing the feasibility of minimal-channel cVEP-BCI systems. However, the authors noted that due to the instability of the calibration, the process occasionally had to be repeated to ensure a minimum calibration accuracy of 85% was reached.

Direct comparisons between standard and low-electrode montages have yet to be systematically assessed in cVEPs. While some studies hint that very minimal montages may be feasible, comparative assessment remains to be conducted. The cVEP-BCI field still lacks a large-scale, online investigation of minimal electrode montages. Therefore, this study directly compares a high-density montage with a realistically minimal configuration, using the well-established CCA classification method to determine what is practical. Our goal is to quantify how performance degrades as electrodes are removed and to identify the limits of electrode minimization. In doing so, we hope to provide actionable guidance for designing more efficient, user-friendly cVEP-BCI systems.

## 2. Materials and Methods

### 2.1. Participants

Thirty-eight participants (22 females, 16 males; mean age 25.9 ± 3.6) took part in this study. All participants provided written informed consent in adherence to the Declaration of Helsinki, and the study received approval from the ethical committee of the medical faculty at the University of Duisburg-Essen (protocol code: 21-10096-BO). Participants could withdraw at any time without providing any reasons. The collected data were stored anonymously for analysis, ensuring confidentiality. Each participant received EUR 20 for participating.

### 2.2. Experimental Protocol

The experiment was conducted in the BCI-Lab of Rhine-Waal University of Applied Sciences (HSRW). First, participants received an information sheet detailing the nature of the experiment. After providing their personal information and written consent, they completed the pre-questionnaire (Table 1), which included questions about their experience with BCI systems, vision prescriptions, and level of tiredness. Subsequently, the electrode cap was applied, participants were further briefed on the procedure and operation of the speller, and the experiment then commenced, as further described in Section 2.4.

Participants completed a structured training phase where they sequentially observed four stimuli across multiple blocks, ensuring exposure to each target under controlled conditions. Each trial lasted 2.1 s, with visual cues guiding their focus, followed by brief pauses before the next trial.

Following these explanations, participants engaged in a preliminary practice phase to familiarize themselves with the speller. During this phase, they chose a five-letter word (such as their name) and practiced selecting letters with the speller. The threshold, gaze shift, and time window settings (described in detail in Section 2.6) were calibrated as necessary during this initial free-spelling practice phase. After the free-spelling practice phase, to counterbalance potential learning effects throughout the session, participants began with different spelling tasks. This assignment was determined by their subject number, which was allocated sequentially based on their order of attendance.

After the familiarization run, the participants were asked to perform two spelling tasks, writing the words “HAVE_FUN” and “PROGRAM”. Between the tasks, participants were allowed to rest. To minimize fatigue-induced bias, the experiment duration was kept under one hour, and therefore the number of spelling tasks was limited to six (two with each electrode setup).

The words for the spelling phase were also selected to maintain a balance in class representation (not including the target with the “UNDO” function). When completing the spelling phase without mistakes, the first target needs to be selected 20 times, the second one 16 times, and the third one 18 times (“HAVE_FUN” and “PROGRAM” are perfectly balanced).

The main experiment involved three rounds (“versions”):

(1) Baseline (16 electrodes). Participants spelled the two words (“HAVE_FUN” and “PROGRAM”), requiring multiple selections in the four-target (three-step speller) interface.
(2) Reduced (no retraining) (six electrodes). After the baseline, eight electrodes were removed (leaving PO3, POz, PO4, O1, Oz, O2). The previously trained spatial filters were kept unchanged (i.e., no retraining). Participants spelled two words again.
(3) Reduced (retrained) (six electrodes). Finally, the system was retrained (a new calibration run) with only the six remaining electrodes. Two new words were spelled under the newly trained model.

If a participant could not achieve any correct selections in a round after several attempts, that round was stopped early. After all spelling tasks, participants completed a post-questionnaire on user experience.

### 2.3. Hardware and Electrodes

Subjects were seated in front of an LCD screen (Asus ROG Swift PG258Q (ASUSTeK Computer Inc., Taipei, Taiwan) resolution: 1920×1080 pixels, vertical refresh rate: 240 Hz) at approximately 80 cm distance and were instructed to avoid unnecessary movements since these affect the quality of the recordings negatively. The computer in use was a Dell Precision Desktop (Dell Inc., Round Rock, TX, USA) with NVIDIA RTX3070 (NVIDIA Corp., Santa Carla, CA, USA) graphics card that operated on Microsoft Windows 10 (21H2) Education running on an Intel processor i9-10900K (3.70 GHz). Ag/AgCl electrodes (EASYCAP GmbH, Herrsching, Germany) were used to acquire the EEG signals. These were easily removable from the cap during recordings, which was essential for quickly reducing the electrode count during the online experiment. Standard abrasive electrolytic electrode gel was applied between the electrodes and the scalp. Impedances were kept below 5 kΩ for a good quality recording. A g.USBamp (Guger Technologies, Graz, Austria) EEG amplifier was utilized for the recording.

**Figure 1:**
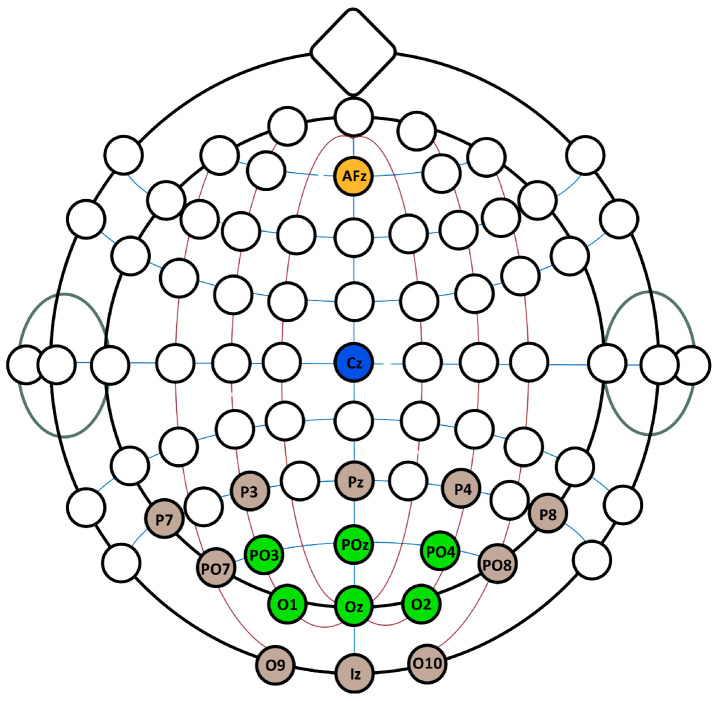
Schematic showing the positions of the electrode positions used. Brown marks the electrodes that are removed after the first two spelling tasks, which only leaves the green electrodes connected. Yellow marks the ground electrode used, while C_z_, marked blue, shows the location of the reference electrode used.

The reference electrode was located at C_Z_ and the ground electrode at AF_Z_. For the first phase of the experiment, sixteen electrodes were used: P7, P3, Pz, P4, P8, PO7, PO3, POz, PO4, PO8, O1, Oz, O2, O9, Iz, and O10, according to the internationally recognized electrode placement system. After the first phase of the experiment, only PO3, POz, PO4, O1, Oz, O2 positions were used, shown in Figure 1 in green.

The decision to select PO3, POz, PO4, O1, Oz, and O2 was based on previous studies [12,18,21,24], which suggest that electrode placement in the occipital and parieto-occipital regions provides optimal coverage for capturing visual evoked potentials (VEPs). These electrodes ensure maximal coverage of the occipital cortex, the primary region responsible for processing visual stimuli. O1 and O2 capture responses from both hemispheres, while Oz records central occipital activity, where cVEP responses typically exhibit the highest amplitude. PO3, POz, and PO4 provide supplementary parieto-occipital coverage, contributing to classification performance in VEP-based BCIs. This selection aims to balance maintaining sufficient signal quality for classification while reducing the complexity of the BCI setup to enhance practical usability. Although reducing the number of electrodes generally affects classification accuracy, this optimal placement could mitigate performance loss.

### 2.4. BCI Speller Interface

**Figure 2:**
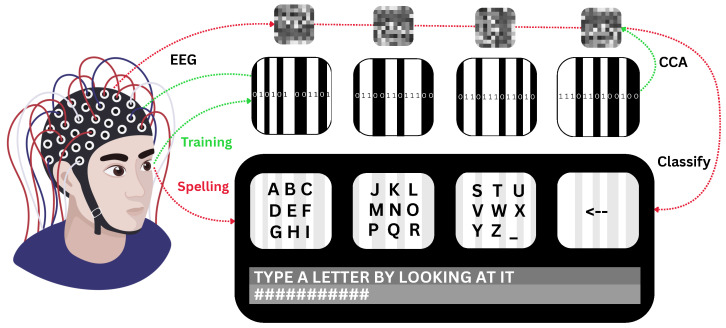
Schematic illustrating the training (green) and spelling (red) phases of the experiment. In the training phase, the participant’s EEG response is recorded while observing the distinct flickering patterns (symbolized by the black and white lines within the boxes) of the four targets, each defined by the m-sequence. Canonical correlation analysis (CCA) is then used to compute spatial filters (visualized at the top of the figure) corresponding to each target. During the spelling phase, the participant fixates on the desired box, and their brain response is spatially filtered using each of the four target-specific filters. The filtered signals are compared to the corresponding stimulus patterns, yielding correlation values, and the target with the highest correlation is selected as the classification result. Upon a correct selection, as the next step in the three-step spelling process, the selected box can split into the letters it contains.

Figure 2 provides an overview of the experimental procedure, illustrating both the training phase, where EEG responses to the flickering targets were recorded and spatial filters were generated via CCA, and the spelling phase, where live classification was performed based on correlation with stimulus patterns.

The experiment utilized a three-step speller interface—implemented in our previous study [17]—that displayed four boxes on the screen: three containing letters (grouped for sequential selection) and one assigned as an “UNDO” option. Following each selection, a brief pause of approximately 2 s was introduced to facilitate gaze shifting before the next input. This layout, which limits the number of simultaneously classified targets, has demonstrated robust and stable performance, as documented in our previous work [17]. Accordingly, employing a system with a proven record of reliability was imperative for ensuring the validity of the subsequent electrode montage comparisons.

During the training phase, participants sequentially observed four distinct stimuli, labeled 1 to 4. The training session consisted of six blocks, denoted as $n_{b}=6$, with each block containing one fixation on each stimulus. This resulted in a total of 6×4=24 trials. Each trial lasted 2.1 s, during which the stimulus pattern was presented for two complete cycles.

A green frame served as a visual cue, guiding the participants by highlighting the specific box they needed to focus on. After each trial, the next target was marked, and the flickering stimulus was momentarily paused for one second. This process was repeated until all six training blocks were completed, after which participants proceeded to the spelling tasks.

During the spelling tasks, the interface displayed four distinct boxes on the screen. Three of these boxes contained grouped letters for selection, while the fourth served as an “UNDO” function. This feature allowed participants to correct errors by either deleting the last entered character or returning to the previous step when needed.

In copy-spelling mode, the system offered immediate visual feedback to indicate selection accuracy. When participants made a correct selection, the corresponding box was highlighted in green, whereas incorrect choices were marked in red. The task required participants to spell predefined words as specified in the experimental protocol (see Section 2.2). To separate words, an underscore character was used in place of spaces. If a mistake was made, participants could utilize the “UNDO” function to either delete the last character or revert to the previous step.

### 2.5. Stimulus Presentation

The spelling interface, also visible in Figure 2, used in this study was adapted from the system outlined in [25]. It displayed four stimulus targets in a 1×4 grid layout. Each box had fixed dimensions of 282×282 pixels to ensure uniform presentation across all trials.

The cVEP stimuli were generated using a 63-bit m-sequence, where a bit value of “0” corresponded to a black state and “1” corresponded to a white state, thereby producing a high-contrast visual stimulus. To produce distinct stimulus sequences for each target class, the base sequence $c_{1}$ was modified via circular shifts. In our experimental setup, we defined K=4 target classes. For each target *k* (with k=1,…,K), the stimulus sequence was obtained by shifting the base sequence $c_{1}$ by (k−1)×4 bits:

(1) $$c_{k}=\mathrm{CircShift}\left(c_{1},\left(k-1\right)\times 4\right)$$

Thus, $c_{2}$ corresponds to $c_{1}$ shifted by 4 bits, $c_{3}$ to a shift of 8 bits, and $c_{4}$ to a shift of 12 bits.

### 2.6. Classification

To translate EEG into target selections, we apply a widely used canonical correlation-based method [5], which learns, for each of the K=4 code-modulated classes, a pair of spatial filters $w_{X,i},w_{Y,i}\in R^{m}$ that maximize the Pearson correlation between the projected EEG and the projected stimulus template. Let *m* be the number of channels and n=2×1.05s×600Hz=1260 the number of samples per trial (two cycles of the 1.05s m-sequence at 600Hz). We record $n_{b}=6$ blocks per class, yielding trials $T_{i}^{j}\in R^{m\times n}$ for class i∈{1,…,4} and block $j\in \{1,\ldots ,n_{b}\}$, and define the class-average response

(2) $${\bar{X}}_{i}=\frac{1}{n_{b}}\sum_{j=1}^{n_{b}}T_{i}^{j}.$$

Meanwhile, $C_{i}\in {\left\{0,1\right\}}^{m\times n}$ is the ideal stimulus template obtained by circularly shifting the base m-sequence by (i−1)×4 bits. CCA then solves

(3) $$\max_{w_{X,i},\;w_{Y,i}}\rho \left(w_{X,i}^{⊤}{\bar{X}}_{i},\;w_{Y,i}^{⊤}C_{i}\right),\;\rho \left(u,v\right)=\frac{u^{⊤}v}{∥u∥∥v∥},$$

yielding one spatial-filter pair per class.

During online spelling, EEG is buffered in blocks of 30 samples (every 0.05 s) into $Y\left(t\right)\in R^{m\times n_{y}}$ until $n_{y}=n$. For each class *i*, we align and extract $R_{i}\left(t\right)$ from $C_{i}$ and compute

(4) $$\lambda_{i}\left(t\right)=\rho \left(w_{X,i}^{⊤}Y\left(t\right),\;w_{Y,i}^{⊤}R_{i}\left(t\right)\right),\;i=1,\ldots ,4.$$

Let $\lambda_{\left(1\right)}$ and $\lambda_{\left(2\right)}$ denote the largest and second largest values of $\left\{\lambda_{i}\left(t\right)\right\}$. We define the certainty margin

(5) $$\Delta C\left(t\right)=\lambda_{\left(1\right)}-\lambda_{\left(2\right)},$$

and issue a selection

(6) $$\hat{i}=\arg\max_{i}\lambda_{i}\left(t\right)$$

only when ΔC(t)>β. Here, β is initialized at 0.15 and hand-tuned between 0.10 and 0.30 during the familiarization run.

Once a selection is made, the buffer Y is cleared, flicker is paused, the chosen target is highlighted for feedback, and a 2 s gaze-shift interval follows before resuming data collection.

### 2.7. Performance Metrics and Statistical Analysis

We quantified BCI performance using two primary metrics:

- Accuracy (%): the ratio of correctly selected targets to total selections (including any corrections via the “UNDO” option).
- Information Transfer Rate (ITR, bits/min): calculated according to Wolpaw et al. as
  (7) $$B=\log_{2}\left(N\right)+P\log_{2}\left(P\right)+\left(1-P\right)\log_{2}\;\left(\frac{1-P}{N-1}\right),$$
  where N=4 is the number of targets, *P* is the observed accuracy, and *B* is then multiplied by selections per minute.

Prior to inferential testing, we assessed normality of the ITR distributions in each condition (Baseline, Reduced no-retrain, Reduced retrain) using the Shapiro–Wilk test.

To account for missing data (participants who failed under some conditions), a linear mixed-effects model was fit with *condition* as a fixed effect and *subject* as a random intercept. We report the overall *F*-test for condition and pairwise contrasts (with Cohen’s *d* and bootstrapped 95% CIs) to compare Baseline vs. Reduced no-retrain, Baseline vs. Reduced retrain, and Reduced no-retrain vs. Reduced retrain.

System functionality rates across conditions were compared with Cochran’s *Q* test, followed by post hoc McNemar tests to identify specific declines (Baseline → Reduced no-retrain, Baseline → Reduced retrain, Reduced no-retrain → Reduced retrain).

To explore whether participant attributes predicted performance or functionality, Pearson correlations were computed between age and ITR in the Reduced-retrained condition, and point-biserial correlations between BCI experience (yes/no) and both ITR and binary retrain success.

Statistical analyses were conducted using Python 3.9.13 (with pandas, SciPy, and statsmodels), Microsoft Excel 2021 (v.1808), and BCI performance calculation tools, available at https://bci-lab.hochschule-rhein-waal.de/en/acc.html (accessed on 2 May 2025).

### 2.8. Questionnaire

Before and after the experiment, participants filled out short questionnaires (Table 1). The pre-questionnaire collected basic information (e.g., BCI experience, vision correction, tiredness). The post-questionnaire assessed perceived flicker disturbance, ease of concentration, and preference for visual feedback style (threshold bar vs. dynamic resizing). Participants also rated how reliable they found the system and whether they could use it daily.

## 3. Results

Across all participants and experimental conditions, the mean classification accuracy was 95.91% (SD = 7.22), while the mean Information Transfer Rate reached 46.49 bits/min (SD = 17.21).

The system functioned successfully for all 38 participants when using the baseline configuration, which incorporated the full set of 16 electrodes. However, a substantial decline in performance was observed when transitioning to the reduced six-electrode setup without retraining the classifier. In this condition, only 18 out of the original 38 participants (47.4%) successfully completed the tasks, underscoring the sensitivity of classification accuracy to spatial electrode coverage.

Introducing classifier retraining in the reduced six-electrode configuration led to a partial recovery, with 23 out of 38 participants (60.5%) successfully completing the tasks. While this improvement suggests that retraining can partially mitigate the performance degradation caused by electrode reduction, it did not fully restore classification performance for all individuals.

The failure rate of the classification pipeline—i.e., the proportion of participants for whom the system, despite repeated trials, was unable to accurately classify the target they were looking at—rose from 0% in the baseline configuration to 52.6% in the reduced (no retraining) condition. With retraining, the failure rate decreased to 39.5%, suggesting that adapting the classifier to the reduced electrode montage improved system usability for some participants.

Among the 20 participants who initially failed in the reduced (no retraining) condition, 7 regained functionality after retraining, while 13 remained unable to perform the tasks. Notably, two participants who had been functional in the reduced (no retraining) condition lost functionality after retraining. This pattern highlights substantial individual differences in neural signal characteristics and sensitivity to electrode placement, which likely influenced classification outcomes.

### 3.1. Version-Wide Results

**Figure 3:**
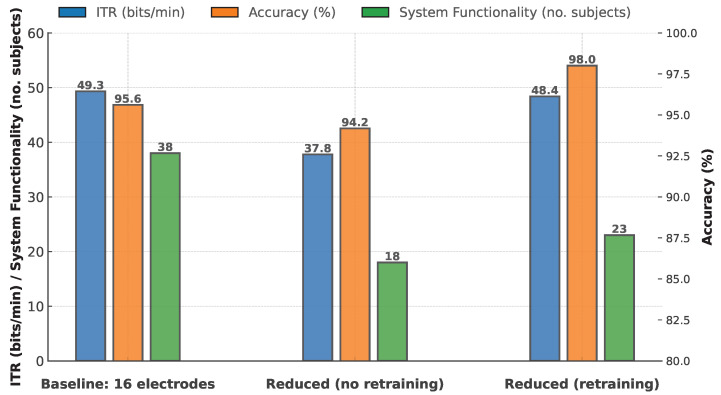
Comparison of ITR, accuracy, and the number of participants for whom the system remained functional across the three experimental rounds (Baseline, Reduced (no retraining), and Reduced (retrained)). The figure visualizes the mean values for each metric, with ITR (bits/min) and number of subjects for whom the system remained functional on the left *y*-axis and accuracy (%) on the right *y*-axis.

Table 2 presents the mean ± SD accuracy and ITR for the three versions: the Baseline setup (16 electrodes), the Reduced setup without retraining (6 electrodes), and the Reduced setup with retraining (6 electrodes). These statistics are computed across all available runs in each version. Figure 3 displays the results alongside system-functionality numbers, which indicate the number of participants for whom the system successfully managed selections under each setup: 38 in the baseline condition, 18 in the reduced condition, and 23 in the reduced-but-retrained condition.

### 3.2. Subject-Specific Results

Table 3 provides the subject-wise performance for all 38 participants. Accuracy is shown in percentages, while ITR is reported in bits/min. A dash (-) indicates that the participant did not produce data for that particular round, typically due to system failure or unsuccessful classification.

### 3.3. Performance Change Across Electrode Configurations

To compare BCI performance (ITR) across the three experimental conditions—Baseline (16 electrodes), Reduced (6 electrodes, no retraining), and Reduced (6 electrodes, retrained)—we conducted statistical analyses. Prior to analysis, a Shapiro–Wilk test was performed to assess the normality of the ITR data. The results confirmed that the data were normally distributed across all conditions (Baseline: W=0.978, p=0.659; Reduced—no-training: W=0.954, p=0.476; Reduced—retrained: W=0.957, p=0.644), satisfying the assumption of normality required for parametric tests.

Given that not all participants completed all experimental conditions, we employed a mixed linear model with subject as a random effect to analyze the impact of electrode configuration on ITR. This model, which accounts for individual variability, revealed a significant effect of condition on ITR (F(2,51.7)=4.74, p=0.013). The estimated mean ITR was 49.33 bits/min for the Baseline condition. In the Reduced (no retraining) condition, ITR was significantly lower by approximately 10.23 bits/min (coefficient = −10.229, p<0.001), whereas in the Reduced (retrained) condition the performance was virtually identical to Baseline (coefficient = 0.128, p=0.956).

Variance component analysis indicated that the fixed effects (electrode configuration) accounted for 6.2% of the total variance (marginal $R^{2}=0.062$), while the combination of fixed and random effects (the latter representing substantial between-subject variability) explained 56.4% of the variance (conditional $R^{2}=0.564$). This suggests that although the electrode configuration exerts a modest direct effect, individual differences play a major role in overall performance.

Pairwise comparisons were performed on the subset of subjects with complete data to further examine the differences among conditions. The comparison between Baseline and Reduced (no retraining) yielded a significant difference (t=2.91, p=0.008) with a large effect size (Cohen’s d=1.177, bootstrapped 95% CI: [0.817, 1.706]). In contrast, the comparison between Baseline and Reduced (retrained) conditions was not statistically significant (t=0.23, p=0.824), with a moderate effect size (Cohen’s d=0.686, 95% CI: [0.411, 1.005]), indicating that retraining restored performance to near baseline levels. Furthermore, the direct comparison between the Reduced (no retraining) and Reduced (retrained) conditions was significant (t=−2.29, p=0.038; Cohen’s d=−0.57, 95% CI: [−0.874, −0.273]), confirming that retraining was essential to mitigate the performance loss caused by electrode reduction.

### 3.4. Questionnaire Results

About 40% of the participants had prior experience with BCI systems, and roughly 62% reported needing corrective lenses (whether worn or not). On average, participants reported having slept about 7.1 h the night before and showed low entry tiredness (mean ≈ 1.68 on a three-point scale). When asked about the system’s flickering, most rated it as only moderately disturbing. In addition, the majority indicated willingness to repeat the experiment and to consider daily use of such a system, with most suggesting that breaks every 1–3 h would be necessary. Finally, most respondents considered a BCI-based interface to be a reliable control method, or at least potentially so. Detailed results of the questionnaire are shown in Table 4.

## 4. Discussion

The reduction in EEG electrodes from sixteen to six in our cVEP-BCI system led to a notable decrease in overall system functionality, even though mean performance metrics for successful users could largely be restored through classifier retraining. This central finding underscores several important considerations for practical BCI design.

Even though no large-scale evaluations were conducted, previous research did not hint at such a high dropout rate in terms of user functionality. Therefore, we conducted additional analyses. The Cochran’s Q test revealed a significant difference in functionality rates across conditions (Q=40.00, p<0.001). The system remained functional for all 38 participants in the Baseline condition but only for 18 participants (47.4%) in the Reduced (no retraining) condition and 23 participants (60.5%) in the Reduced (retrained) condition. Post hoc McNemar tests confirmed a significant decline in functionality from Baseline to Reduced (no retraining) ($\chi^{2}=20.00$, p<0.001) and from Baseline to Reduced (retrained) ($\chi^{2}=15.00$, p<0.001). Although the improvement in functionality from Reduced (no retraining) to Reduced (retrained) showed an increase from 18 to 23 participants, this difference did not reach statistical significance ($\chi^{2}=2.27$, p=0.132).

The fact that, with retraining, the system achieved performance levels statistically indistinguishable from the baseline —despite a significant reduction in the number of participants for whom the system remained functional— may be due to individual differences. Potentially important channels may have been dropped (and this fact was previously never properly analyzed), but if enough remained, the system continued to function, and performance improved with retraining. Other factors, such as the known sensitivity of CCA to electrode position shifts, could also contribute to this outcome [26].

We also examined whether participant age and prior BCI experience were associated with (1) ITR in the Reduced (retrained) condition and (2) the probability of a functional system after retraining. Pearson’s correlation between age and ITR in the Reduced (retrained) condition was small and non-significant (r=−0.148, p=0.511), indicating no clear effect of age on retrained performance. A point-biserial correlation between prior BCI experience and ITR in the Reduced (retrained) condition yielded a moderate positive coefficient (r=0.337) that likewise failed to reach significance (p=0.125), suggesting only a non-significant trend for experienced users to achieve higher ITR. Crucially, neither age (r=−0.115, p=0.500) nor prior BCI experience (r=−0.103, p=0.544) significantly predicted whether the system remained functional after retraining, showing that individual differences in final system functionality cannot be explained by age or prior BCI exposure.

It is important to emphasize that non-functionality could, in some cases, be solely due to lower SNR and possibly higher electrode impedance, which may have caused the classification to get stuck and never fully reach the required certainty threshold (β) (applied to ΔC(t), see Equation (5)). However, the scale of this phenomenon suggests that the issue is not merely due to a reduction in the signal-to-noise ratio, but also that, for some participants, the crucial electrode locations required to capture their unique cVEP response patterns were eliminated with the electrode reduction. Supporting the idea of pronounced inter-individual variability, recent work has demonstrated that these personalized cVEP response patterns may even be used for individual identification [27]. Furthermore, classification methods that allow for continuous refinement or adaptive updating may achieve better performance, given that a short training period may not fully capture the evolving spatial and temporal characteristics observed during prolonged use—an effect that is amplified when fewer electrodes are available. This limitation of the CCA classification method is underscored by the fact that two participants who initially had a functioning system lost functionality after retraining, highlighting the need for improved approaches that can better adapt to reduced electrode configurations.

### Future Research Directions

To address the challenges of individual variability with reduced electrode montages, future research should prioritize the development and evaluation of adaptive and personalized BCI systems while also placing greater emphasis on advanced signal processing techniques. It would also be interesting to conduct more large-scale investigations on cVEP response spatial localization and its individual differences.

Subsequent studies could further explore advanced artifact rejection, denoising, adaptive filtering, and deep autoencoder-based cleaning techniques [28,29,30] to mitigate the impact of eye movements and movement artifacts. Integrating these preprocessing methods with adaptive classification pipelines will be essential to enhance the robustness of low-electrode montages.

Future experimental designs should focus on real-time electrode selection and refinement. Developing systems capable of dynamically assessing electrode importance would enable the progressive elimination of non-essential electrodes during operation. This approach could offer clearer insights into inter-individual differences and quantify performance impacts due to electrode reduction. Personalized optimal electrode subsets could be identified using methods such as sequential feature selection [31] or mutual information analysis [32]. These techniques highlight the most informative electrodes for each individual, ensuring robust performance with minimal electrode counts. Additionally, online adaptive electrode selection methods employing reinforcement learning or attention mechanisms should be explored to dynamically optimize electrode configurations.

To enhance performance across all users with minimal montages, classification methods more robust than CCA should be considered. Convolutional neural networks (CNNs) [33], hybrid CNN-RNN models [14], and spatiotemporal convolutional networks (ST-CNNs) [34] are promising alternatives, as they effectively capture temporal dynamics and spatial relationships within EEG signals, potentially enhancing accuracy even with fewer electrodes. Incorporating adaptive classifiers that dynamically adjust their parameters or structures based on real-time, user-specific EEG responses could further enhance these models. Additionally, employing transfer learning—where models pre-trained on larger or related datasets rapidly adapt to individual users [35]—may significantly reduce calibration time and performance variability across users.

Graph Neural Networks (GNNs), particularly Graph Attention Networks (GATs), hold significant promise for modeling spatial dependencies in EEG data [36]. By representing electrodes as nodes in a graph, GNNs can effectively learn inter-electrode relationships and identify critical sensor locations, potentially guiding adaptive electrode reduction strategies. Future research should explore the application of GNNs to cVEP-BCI for identifying optimal spatial features and improving personalized electrode selection.

In summary, this study demonstrates both the potential and the limitations of using a standardized, minimal electrode montage for cVEP-based BCIs with the classic CCA approach. It provides a guideline for how far—and to what degree—electrodes could be reduced without sacrificing performance. On the one hand, it identifies the point at which dynamic, personalized approaches can be essential for achieving consistently high performance with only a few electrodes; on the other hand, it points toward alternative strategies when the goal is to increase user-friendliness or reduce equipment cost. Ultimately, this work aims to offer a clear benchmark for one of the key avenues toward making cVEP-BCI systems more practical.

## Figures and Tables

**Table 1:** Overview of pre- and post-experiment questionnaires.

Pre/Post Experiment	Questions
**Pre**	Have you ever used a BCI system? If yes, please describe briefly. Do you have a vision prescription? If yes, are you wearing it now? How tired do you feel right now? (1 = not at all, 6 = very much) How many hours did you sleep last night?
**Post**	How tired do you feel now? (1 = not at all, 6 = very much) Did you find the flickering disturbing? (1 = not at all, 6 = very much) Was it easy to concentrate on the boxes? (1 = not at all, 6 = very much) Would you repeat the experiment? (Yes/No/Maybe) Could you use the system daily? (Yes/No/Maybe) How long do you think one could use the system without breaks? Do you consider BCI a reliable control method? (Yes/No/Maybe)

**Table 2:** Mean ± SD of accuracy and ITR for each experimental round (version).

Round	Accuracy (%)	ITR (bits/min)
Baseline Setup (16 electrodes)	95.62 ± 8.31	49.33 ± 17.07
Reduced Setup (no retraining) (6 electrodes)	94.18 ± 8.00	37.79 ± 18.68
Reduced Setup (retrained) (6 electrodes)	98.01 ± 3.21	48.39 ± 14.24

**Table 3:** Subject-wise accuracy (%) and ITR (bits/min) across the three versions. Red stands for Reduced. A dash (-) indicates no data for that round.

Subject	Accuracy (%)			ITR (bits/min)		
	Baseline	Red. (No Retrain)	Red. (Retrain)	Baseline	Red. (No Retrain)	Red. (Retrain)
1	89.51	95.90	96.00	29.38	15.63	32.41
2	100.00	73.56	100.00	52.77	14.22	35.12
3	100.00	-	-	54.14	-	-
4	89.71	-	98.00	42.40	-	51.72
5	98.00	100.00	98.00	62.63	48.99	68.25
6	100.00	-	-	58.46	-	-
7	90.00	96.08	-	23.31	38.25	-
8	92.60	-	94.83	37.40	-	35.18
9	80.52	-	100.00	28.37	-	47.70
10	96.66	-	-	64.25	-	-
11	100.00	-	-	43.85	-	-
12	100.00	-	-	67.01	-	-
13	96.08	-	-	23.28	-	-
14	98.08	-	-	53.19	-	-
15	100.00	-	-	72.16	-	-
16	91.03	83.53	97.83	46.65	21.58	54.91
17	96.43	-	-	55.81	-	-
18	100.00	-	-	67.11	-	-
19	100.00	-	100.00	63.29	-	50.17
20	98.08	-	96.43	63.14	-	51.78
21	100.00	100.00	97.83	57.30	54.49	37.97
22	100.00	-	100.00	56.59	-	56.02
23	100.00	89.77	98.00	35.98	29.23	41.04
24	92.65	97.83	100.00	30.38	24.20	39.49
25	100.00	100.00	100.00	53.12	48.10	52.70
26	90.18	95.90	96.00	59.43	67.43	77.57
27	100.00	100.00	97.83	74.90	75.51	70.38
28	100.00	100.00	100.00	41.80	32.04	41.04
29	82.36	86.17	94.45	30.59	25.89	31.51
30	100.00	100.00	100.00	50.74	43.19	49.20
31	100.00	-	98.22	35.73	-	23.97
32	100.00	98.00	98.08	46.47	32.79	50.29
33	73.88	95.90	92.73	26.24	58.17	56.03
34	100.00	96.30	100.00	76.10	27.36	58.54
35	97.83	-	-	35.17	-	-
36	88.85	78.38	-	37.04	8.39	-
37	100.00	-	-	69.76	-	-
38	91.00	-	-	48.48	-	-

**Table 4:** Summary of questionnaire results (N = 38).

Characteristic	Value/Distribution
Age (years)	Mean ≈ 25.9
Gender	∼58% Female, ∼42% Male
Prior BCI experience	15 Yes (39%), 23 No (61%)
Vision prescription needed	21 Yes (55%), 17 No (45%)
Hours slept last night	Mean ≈ 7.1 h
Entry tiredness level	Mean ≈ 1.68 (6-point scale)
Flickering disturbance rating	Mean ≈ 3.18 on a 6-point scale (Moderately disturbing)
Willingness to repeat experiment	∼50% Yes, ∼30% Maybe, ∼20% No
Adoption for daily use	∼50% Yes, ∼30% Maybe, ∼20% No
Estimated possible continuous use duration	Mean ≈ 2.09 h without break
BCI as a reliable control method	∼60% Yes, ∼30% Maybe, ∼10% No

## Data Availability

The availability of these data is restricted. Data were stored anonymously for analysis purposes only to ensure participant confidentiality. Ethical approval did not cover the publication of EEG data, so the data cannot be shared.

## Abbreviations

The following abbreviations are used in this manuscript:

BCI	Brain–Computer Interface
cVEP	Code-Modulated Visual Evoked Potential
EEG	Electroencephalography
ITR	Information Transfer Rate
CCA	Canonical Correlation Analysis
VEP	Visual Evoked Potential
SSVEP	Steady-State Visual Evoked Potential
ERP	Event-Related Potential
SNR	Signal-to-Noise Ratio
ANOVA	Analysis of Variance
CNN	Convolutional Neural Network
GNN	Graph Neural Network
GAT	Graph Attention Network
SVM	Support Vector Machine
PCA	Principal Component Analysis
ICA	Independent Component Analysis
AFz	Anterior Frontal Midline (Reference Electrode)
Cz	Central Midline (Reference Electrode)
POz	Parieto-Occipital Midline (Electrode Position)
Hz	Hertz (Frequency Unit)
ms	Milliseconds
